# Myocardial microvascular function at rest and stress measured with dynamic contrast-enhanced MRI

**DOI:** 10.1186/1532-429X-14-S1-P283

**Published:** 2012-02-01

**Authors:** David A Broadbent, John D Biglands, Abdulghani M Larghat, Steven Sourbron, Sven Plein, David L Buckley

**Affiliations:** 1 Medical Physics, Leeds Teaching Hospitals NHS Trust, Leeds, UK; 2 Division of Medical Physics, University of Leeds, Leeds, UK; 3 Division of Cardiovascular and Neuronal Remodelling, University of Leeds, Leeds, UK

## Summary

Dynamic contrast-enhanced (DCE) MRI was performed in 16 healthy subjects to measure myocardial microvascular characteristics. In addition, repeatability was assessed in 11 of those subjects who underwent a second examination. The results showed that Gd-DTPA transport in the myocardium is not perfusion limited at rest or under adenosine stress.

## Background

DCE MRI has been used to estimate myocardial perfusion (blood flow, Fb) but there has been little work published on using this technique to measure related microvascular characteristics, such as microvessel permeability-surface area product (PS) and interstitial volume (extra-vascular extra-cellular volume, ve). The reason may be that transport of Gd-DTPA in resting myocardium is thought to be perfusion limited (with near complete first pass extraction). This study aimed to assess the potential of measuring microvascular characteristics in healthy individuals at rest and under adenosine stress and to assess the repeatability of the measurements.

## Methods

16 healthy volunteers (9 males, age 34±8 years) underwent rest and adenosine stress DCE MRI study, using Gd-DTPA (Magnevist, Bayer Schering Pharma, Berlin, Germany), with informed consent. 11 (6 males, age 33±7) returned for repeat examination. A single slice, breath-hold, saturation recovery segmented gradient echo sequence was used (Radjenovic A et al. 2010, Magn Reson Med 64 pp1696-1703). Endo- and epicardial contours were traced with manual correction for respiratory motion. The arterial input function was derived from the left ventricular cavity. Signal-time courses were converted to concentration-time courses according to Biglands J et al. 2011 (Phys Med Biol 56 pp2423-2443). Concentration-time data were analyzed using a distributed parameter tracer kinetic model (Larson K et al. 1987, J Cereb Blood Flow Metab 7 pp443-463) with Laplace domain fitting (Garperbring A et al. 2009, IEEE Trans Med Imaging 28 pp1375-1383) to estimate Fb, myocardial perfusion reserve (MPR), first-pass extraction fraction (E), blood volume (vb), PS and ve. Within-subject coefficient of variation (wCV) was calculated to assess repeatability, which was compared to that achieved using the Fermi model (Jerosch-Herold M et al. 1998, Med Phys 25 pp73-84) to measure Fb.

## Results

Adenosine stress induced significant increases in Fb (1.5±0.6 to 3.6±1.2 ml/min/ml tissue, rest to stress, MPR = 2.6±1.1), vb (9±7 to 13±3 %) and PS (0.9±0.4 to 1.8±1.1 ml/min/ml tissue), all p<0.05. There was negligible effect on ve (17±5 to 17±3 %) or E (0.60±0.20 to 0.56±0.14). In the repeatability assessment wCV values were 31/23 % (rest/stress) for Fb and 18/28 % for the Fermi model fit. MPR wCV was 31 % (distributed parameter model) and 34 % (Fermi model). For the additional parameters from the distributed parameter model wCV was 76/27 % for vb, 45/42 % for PS, 18/12 % for ve and 27/28 % for E.

## Conclusions

Myocardial Gd-DTPA transport is not perfusion limited and the use of DCE MRI to measure characteristics such as permeability-surface area product and interstitial volume is feasible. Use of the distributed parameter model to extract these measurements show comparable repeatability compared with estimates of blood flow with the Fermi model.

## Funding

Supported in part by the British Heart Foundation (RG/05/004).

